# Easy NanoString nCounter data analysis with the NanoTube

**DOI:** 10.1093/bioinformatics/btac762

**Published:** 2022-11-28

**Authors:** Caleb A Class, Caiden J Lukan, Christopher A Bristow, Kim-Anh Do

**Affiliations:** Department of Pharmaceutical Sciences, Butler University, Indianapolis, IN 46208, USA; Department of Pharmaceutical Sciences, Butler University, Indianapolis, IN 46208, USA; TRACTION, The University of Texas MD Anderson Cancer Center, Houston, TX 77030, USA; Department of Biostatistics, The University of Texas MD Anderson Cancer Center, Houston, TX 77030, USA

## Abstract

**Summary:**

The NanoTube is an open-source pipeline that simplifies the processing, quality control, normalization and analysis of NanoString nCounter gene expression data. It is implemented in an extensible R library, which performs a variety of gene expression analysis techniques and contains additional functions for integration with other R libraries performing advanced NanoString analysis techniques. Additionally, the NanoTube web application is available as a simple tool for researchers without programming expertise.

**Availability and implementation:**

The NanoTube R package is available on Bioconductor under the GPL-3 license (https://www.bioconductor.org/packages/NanoTube/). The R-Shiny application can be downloaded at https://github.com/calebclass/Shiny-NanoTube, or a simplified version of this application can be run on all major browsers, at https://research.butler.edu/nanotube/.

**Supplementary information:**

Supplementary data are available at *Bioinformatics* online.

## 1 Introduction

The NanoString nCounter gene expression analysis system has become a popular method for gene expression profiling studies in a variety of research areas (Veldman-Jones *et al.*, 2015). By directly measuring gene expression without cDNA preparation and polymerase chain reaction (PCR) analysis, this system can streamline sample preparation and expression profiling when up to 800 targets are to be analyzed—for example, in validation studies or when the researcher wants to focus on a certain gene type (Geiss *et al.*, 2008). However, the data analysis step can still be an obstacle, requiring the use of proprietary analysis software or an analyst experienced in data processing and normalization, as well as the selection and implementation of the proper differential expression analysis method. Previous R libraries have tackled certain steps of the nCounter analysis pipeline, including quality control, normalization and differential expression analysis, but an all-in-one, the open-source package has yet to be available (Canouil *et al.*, 2020; Waggott *et al.*, 2012; Wang *et al.*, 2016).

In this article, we introduce the NanoTube, an open-source R library (available via Bioconductor) including functions for all steps of NanoString nCounter gene expression data analysis, as well as integration with other R libraries for NanoString data analysis (Huber *et al.*, 2015; Molania *et al.*, 2019; Wang *et al.*, 2016). Additionally, we present a web application for data processing and analysis (built using R-Shiny), which performs this pipeline for researchers without R programming experience (Sievert, 2020).

## 2 Methods and features

A brief summary of the NanoTube’s functions and features is provided in this section. Much more detail, as well as usage examples, can be found in the Bioconductor vignette, and the R library and web application’s help files.

### 2.1 The R library

The R version of the NanoTube can process data from Reporter Code Count files, or from tabular files containing raw counts of all reporters (subsequently referred to as ‘genes’) in all samples. A sample metadata file can also be loaded at this stage, and the full dataset will be saved as an ExpressionSet object. Basic quality control procedures are then conducted, including the correlation of observed versus expected counts for positive control genes, as well as a summary of negative control genes.

NanoString data can then be normalized by one of three methods. The standard method involves within-sample normalization using positive control reporters and housekeeping genes, as well as the removal of endogenous genes found to be at or below the level of ‘noise’, which is calculated using the observed values of the negative control genes (Waggott *et al.*, 2012). Alternatively, the Removing Unwanted Variation-III (RUV-III) method has been demonstrated to provide improved performance for datasets including technical replicates—particularly when those technical replicates span multiple batches—and it can also be used for normalization without true replicates by generating pseudoreplicates from pseudosamples (Molania *et al.*, 2019, 2022). Finally, the RUVg method has been demonstrated on multiple NanoString datasets to provide improved normalization performance using housekeeping genes (Bhattacharya *et al.*, 2020; Risso *et al.*, 2014). Any of these methods (‘nSolver’, ‘RUVIII’, ‘RUVg’ or ‘none’ for no normalization) can be selected using the ‘normalization’ option in processNanostringData, and they can be further tuned and customized using additional options described in the Bioconductor vignette. Principal component analysis can be used to assess the normalization performance with the nanostringPCA function, in addition to relative log expression (RLE) plots using the ruv_rle function from the RUV library (Molania *et al.*, 2019).

By default, differential expression analysis is conducted after normalization using the limma library (Ritchie *et al.*, 2015). The NanoTube automatically conducts simple 2-group comparisons based on sample group information provided by the user, but more complex analyses can be done by directly supplying a design matrix. As an alternative to limma, the NanoStringDiff method can directly be applied on the raw data after converting to the proper format using the makeNanoStringSetFromEset function (Wang *et al.*, 2016). This method applies a generalized linear model of the negative binomial family, and it has been shown to outperform other methods in simulation studies, at the cost of being much more computationally intensive. A brief vignette is provided in the Supplementary Materials, comparing the use of the two differential expression methods in the NanoTube on two real datasets of different sizes (*n* = 3 and *n* = 14 samples per group).

Gene set enrichment analysis can be performed with the results from either differential expression method, using the pre-ranked FGSEA implementation in R (Subramanian *et al.*, 2005). This step only considers genes that were above the detection limit and analyzed by differential expression analysis, to avoid introducing bias based on the selected NanoString gene panel. Additional functions for post-analysis steps, including leading-edge analysis and similarity clustering of enriched gene sets (based on the fraction of leading edge genes shared between sets), are provided to facilitate the easier interpretation of gene set analysis results.

### 2.2 The R-Shiny application

**Fig. 1.**
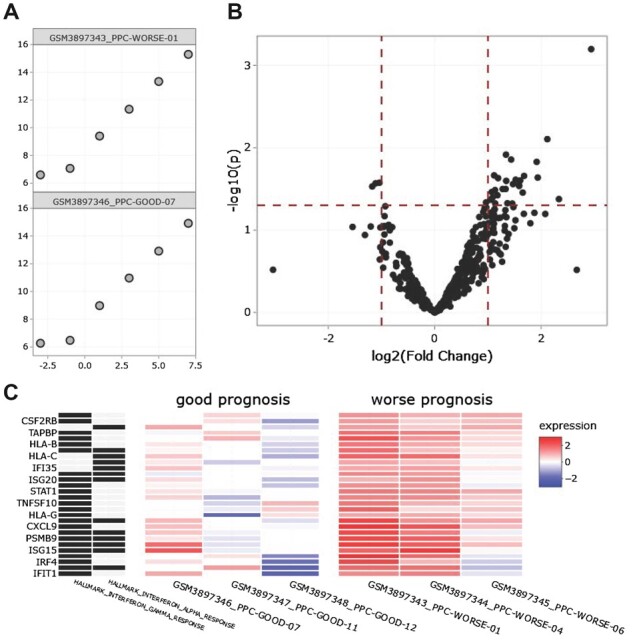
A sampling of visualizations generated by the NanoTube web app. These plots were built using the plotly library to allow interactivity. Data from GSE132946 (D’Angelo *et al.*, 2019) were used to generate figures. The MSigDB Hallmark database was used for gene set analysis (Liberzon *et al.*, 2011). (**A**) Observed versus expected log2-counts of positive control target detection in each sample (cropped to show subset of samples). (**B**) Volcano plot of ‘Worse’ versus ‘Good’ prognosis group. Gene symbols can be viewed by hovering over individual points. (**C**) Heatmaps are built based on the clustered GSEA results. Pathway membership of each gene is provided in the 2-column heatmap on the left, and across-sample gene expression is presented in the heatmap on the right

The NanoTube web application was developed using R-Shiny, and it provides a graphical user interface to simplify all steps in the NanoString gene expression analysis workflow (Sievert, 2020). Initial data-checking allows the user to confirm the proper input of their expression data and sample information table prior to processing. A design matrix can also be uploaded to facilitate multivariate analysis. Visualizations provided include QC plots, principal component analysis, DE volcano plot and heatmaps, as well as relevant result data tables. Many of the visualization outputs are generated using the plotly library, which better allows the user to interpret and understand their own data (Fig. 1).

## Supplementary Material

btac762_Supplementary_Data
