# Supplementary material for: Easy NanoString nCounter data analysis with the NanoTube
Source: Bioinformatics. 2022 Nov 28;39(1):btac762. doi: 10.1093/bioinformatics/btac762 (PMC9805552; doi:10.1093/bioinformatics/btac762)
Supplement: btac762_Supplementary_Data [file btac762_supplementary_data.zip › btac762_Supplementary_Data/NanoTube-Limma_and_NanoStringDiff.pdf]

# A brief comparison of NanoStringDiff and Limma analysis with the NanoTube

Caleb A. Class

## Introduction

The NanoString nCounter (R) platform includes a variety of controls to allow for accurate quantification of endogenous target ("gene") expression. This includes Positive Controls with an expected expression level, Negative Controls with no expected expression, and Housekeeping Genes that are expected to have consistent expression across samples. Traditionally, positive control and housekeeping genes were used to scale the expression levels of endogenous genes, while negative controls were used as the "noise" level: this level was either subtracted from endogenous genes, or used to remove endogenous genes from analysis if their overall expression was below this level (Waggott et al. 2012). After this normalization, differential expression analysis was conducted using a method based on t tests. Alternatively, Wang et al. proposed a method, NanoStringDiff, which uses a linear model of the negative binomial family, including all of the control genes in the model to improve the estimation of differential expression (Wang et al. 2016). NanoStringDiff was demonstrated using simulation data, and one real data set, to substantially outperform the scaling normalization and t-test method, particularly when sample sizes were small or when many genes have expressions near noise level.

The NanoTube analysis package provides two main options for differential expression analysis. Traditional normalization can be conducted by scaling based on positive control and housekeeping genes, as well as subtracting noise levels and/or removing endogenous genes with low expression compared to the calculated noise level; after these steps (particularly when low-expression genes are removed), expression distributions generally resemble a normal distribution, and differential expression analysis can then be conducted using Limma (Ritchie et al. 2015). Alternatively, data can be loaded using NanoTube and then analyzed in NanoStringDiff. Both methods allow subsequent gene set analysis using NanoTube functions.

The purpose of this vignette is three-fold: to directly compare differential expression results using scaling normalization and Limma vs. NanoStringDiff for two real data sets, to profile the computational time taken by the two methods, and to demonstrate the implementation of the two methods in the NanoTube. This vignette does not aim to reach a conclusive verdict regarding the two methods, but to provide a few tips based on what we've seen so far.

## Analysis

### A smaller data set

First, we'll load the data from Horbinski data set, which was generated and used to test NanoStringDiff, both in a real data analysis and to assist in generating their simulation data sets (Wang et al. 2016). This data set contains 6 samples (3 per group), comparing human glioma cells with a certain IDH1 mutation vs. those without.

We'll load the data in four ways, as described in the comments.

```
knitr::opts_chunk$set(tidy = FALSE,
                      cache = FALSE,
                      message = FALSE, error = FALSE, warning = TRUE,
```

```
                      fig.height = 3.5, fig.width = 3.5)

library(NanoTube)

# No normalization, to use in NanoStringDiff
datNoNorm <- processNanostringData("../../extra_data/GSE80821_RAW.tar",
                             sampleTab = "../../extra_data/GSE80821_sample_data.csv",
                             idCol = "Sample", groupCol = "Group",
                             normalization = "none")

# Use scaling normalization for positive and housekeeping controls,
# but do not use negative control genes.
datKeep <- processNanostringData("../../extra_data/GSE80821_RAW.tar",
                             sampleTab = "../../extra_data/GSE80821_sample_data.csv",
                             idCol = "Sample", groupCol = "Group",
                             bgProportion = 0) # bgProportion = 0 lets us keep all genes

# Use default scaling normalization, and remove genes that do
# not exceed "noise" level (mean + 2sd of negative controls) in at least half of the samples.
datDefault <- processNanostringData("../../extra_data/GSE80821_RAW.tar",
                             sampleTab = "../../extra_data/GSE80821_sample_data.csv",
                             idCol = "Sample", groupCol = "Group")

# Provides basic QC parameters
datQC <- processNanostringData("../../extra_data/GSE80821_RAW.tar",
                             sampleTab = "../../extra_data/GSE80821_sample_data.csv",
                             idCol = "Sample", groupCol = "Group",
                             output.format = "list")
```

Check that the RCC files and sample information (CSV) file were merged correctly. The sample names match the rownames, so it's ok.

```
knitr::kable(pData(datNoNorm),
            row.names = TRUE, align = "c")
```

| | Sample | Group | groups | normalization |
|---|---|---|---|---|
| GSM2138338_20130423_Horbinski-042313-1-12_GFP-1_06.RCC.gz | GSM2138338_20130423_Horbinski-042313-1-12_GFP-1_06.RCC | GFP-vector | GFP-vector | none |
| GSM2138339_20130423_Horbinski-042313-1-12_GFP-2_07.RCC.gz | GSM2138339_20130423_Horbinski-042313-1-12_GFP-2_07.RCC | GFP-vector | GFP-vector | none |
| GSM2138340_20130423_Horbinski-042313-1-12_GFP-3_08.RCC.gz | GSM2138340_20130423_Horbinski-042313-1-12_GFP-3_08.RCC | GFP-vector | GFP-vector | none |
| GSM2138341_20130423_Horbinski-042313-1-12_mutant-1_09.RCC.gz | GSM2138341_20130423_Horbinski-042313-1-12_mutant-1_09.RCC | GFP-R132H-IDH1 | GFP-R132H-IDH1 | none |
| GSM2138342_20130423_Horbinski-042313-1-12_mutant-2_10.RCC.gz | GSM2138342_20130423_Horbinski-042313-1-12_mutant-2_10.RCC | GFP-R132H-IDH1 | GFP-R132H-IDH1 | none |
| GSM2138343_20130423_Horbinski-042313-1-12_mutant-3_11.RCC.gz | GSM2138343_20130423_Horbinski-042313-1-12_mutant-3_11.RCC | GFP-R132H-IDH1 | GFP-R132H-IDH1 | none |

Check QC scale factors. These are expected to be between 0.3 and 3.

Positive control scale factors are ok.

```
signif(datQC$pc.scalefactors, digits = 2)
```

```
##    GSM2138338_20130423_Horbinski-042313-1-12_GFP-1_06.RCC.gz
##                                                         0.77
##    GSM2138339_20130423_Horbinski-042313-1-12_GFP-2_07.RCC.gz
##                                                         1.20
##    GSM2138340_20130423_Horbinski-042313-1-12_GFP-3_08.RCC.gz
##                                                         1.20
## GSM2138341_20130423_Horbinski-042313-1-12_mutant-1_09.RCC.gz
##                                                         0.93
## GSM2138342_20130423_Horbinski-042313-1-12_mutant-2_10.RCC.gz
##                                                         1.10
## GSM2138343_20130423_Horbinski-042313-1-12_mutant-3_11.RCC.gz
##                                                         0.81
```

Housekeeping scale factors are also ok.

```
signif(datQC$hk.scalefactors, digits = 2)
```

```
##    GSM2138338_20130423_Horbinski-042313-1-12_GFP-1_06.RCC.gz
##                                                         0.75
##    GSM2138339_20130423_Horbinski-042313-1-12_GFP-2_07.RCC.gz
##                                                         0.95
##    GSM2138340_20130423_Horbinski-042313-1-12_GFP-3_08.RCC.gz
##                                                         1.10
## GSM2138341_20130423_Horbinski-042313-1-12_mutant-1_09.RCC.gz
##                                                         1.30
## GSM2138342_20130423_Horbinski-042313-1-12_mutant-2_10.RCC.gz
##                                                         0.86
## GSM2138343_20130423_Horbinski-042313-1-12_mutant-3_11.RCC.gz
##                                                         1.10
```

Most genes in this data set are expressed at a lower level than "background", which is calculated as the mean plus two standard deviations of the negative controls. This can be seen in the last column of the table below, which shows the fraction of genes with lower expression than this background threshold in each sample. 597 of 822 genes are discarded from this data set with the default filtering.

```
bgTab <- datQC$bg.stats

bgTab[,c(1:4,6)] <- lapply(bgTab[,c(1:4,6)], format, digits = 1, nsmall = 1)
knitr::kable(bgTab,
             row.names = TRUE, align = "c")
```

| | Mean.Neg | Max.Neg | sd.Neg | background | num.less.bg | frc.less.bg |
|---|---|---|---|---|---|---|
| GSM2138338_20130423_Horbinski-042313-1-12_GFP-1_06.RCC.gz | 10.8 | 18.1 | 4.9 | 20.6 | 590 | 0.7 |
| GSM2138339_20130423_Horbinski-042313-1-12_GFP-2_07.RCC.gz | 8.4 | 11.5 | 2.5 | 13.4 | 414 | 0.5 |
| GSM2138340_20130423_Horbinski-042313-1-12_GFP-3_08.RCC.gz | 9.2 | 12.3 | 1.7 | 12.5 | 360 | 0.4 |
| GSM2138341_20130423_Horbinski-042313-1-12_mutant-1_09.RCC.gz | 14.4 | 20.5 | 4.9 | 24.1 | 683 | 0.9 |

| | Mean.Neg | Max.Neg | sd.Neg | background | num.less.bg | frc.less.bg |
|---|---|---|---|---|---|---|
| GSM2138342_20130423_Horbinski-042313-1-12_mutant-2_10.RCC.gz | 11.3 | 16.0 | 5.2 | 21.7 | 674 | 0.8 |
| GSM2138343_20130423_Horbinski-042313-1-12_mutant-3_11.RCC.gz | 10.3 | 22.3 | 7.2 | 24.7 | 687 | 0.9 |

Next, conduct Limma analysis with the default scaling options and no usage of negative control genes. The low expression genes are not expected to conform to a normal distribution, so this is not generally recommended. In this case, including all genes will allow us to compare results of all genes between the two methods. 'end_time - start_time' (displayed below the chunk) shows that Limma analysis was quite fast on an HP laptop with 2 x 16 MB memory.

```
start_time <- Sys.time()

res.lim <- runLimmaAnalysis(datKeep, base.group = "GFP-vector")

end_time <- Sys.time()
signif(end_time - start_time, digits = 1)
```

```
## Time difference of 0.05 secs
```

```
# Retain results for Mutant vs. Control
res.lim <- topTable(res.lim, number = nrow(res.lim), coef = 2)
```

Prepare the data set for NanoStringDiff analysis.

```
dat.ns <- makeNanoStringSetFromEset(datNoNorm)

dat.ns <- NanoStringDiff::estNormalizationFactors(dat.ns)

knitr::kable(head(pData(dat.ns)), row.names = TRUE, align = "c")
```

| | GFP-R132H-IDH1 | GFP-vector |
|---|---|---|
| GSM2138338_20130423_Horbinski-042313-1-12_GFP-1_06.RCC.gz | 0 | 1 |
| GSM2138339_20130423_Horbinski-042313-1-12_GFP-2_07.RCC.gz | 0 | 1 |
| GSM2138340_20130423_Horbinski-042313-1-12_GFP-3_08.RCC.gz | 0 | 1 |
| GSM2138341_20130423_Horbinski-042313-1-12_mutant-1_09.RCC.gz | 1 | 0 |
| GSM2138342_20130423_Horbinski-042313-1-12_mutant-2_10.RCC.gz | 1 | 0 |
| GSM2138343_20130423_Horbinski-042313-1-12_mutant-3_11.RCC.gz | 1 | 0 |

Run NanoStringDiff analysis. This was much slower on the same HP laptop, but still manageable with the smaller sample size. This method is particularly computationally intense, particularly due to inclusion of the negative control data in the generalized linear model (Wang et al. 2016).

```
start_time <- Sys.time()
```

```r
res.ns <- NanoStringDiff::glm.LRT(dat.ns,
                                  design.full = as.matrix(pData(dat.ns)),
                                  contrast = c(1, -1))  # IDH1 vs. vector

end_time <- Sys.time()
signif(end_time - start_time, digits = 1)
```

```
## Time difference of 3 mins
```

```r
nsTab <- head(res.ns$table[order(res.ns$table$pvalue),])
nsTab[,1:4] <- lapply(nsTab[,1:4], format, digits = 1, nsmall = 1)

knitr::kable(nsTab,
             row.names = TRUE, align = "c")
```

| | logFC | lr | pvalue | qvalue |
|---|---|---|---|---|
| hsa-miR-145-5p\|0 | -1.8 | 87.8 | 0e+00 | 0e+00 |
| hsa-miR-374a-5p\|0 | -1.2 | 51.6 | 7e-13 | 3e-10 |
| hsa-miR-181a-5p\|0 | -1.0 | 37.5 | 9e-10 | 2e-07 |
| hsa-miR-221-3p\|0 | -1.1 | 35.3 | 3e-09 | 6e-07 |
| hsa-miR-151a-3p\|0 | -1.4 | 28.6 | 9e-08 | 1e-05 |
| hsa-miR-374b-5p\|0 | -1.2 | 26.2 | 3e-07 | 4e-05 |

We'll merge the resulting data frame from each analysis by the target name.

```r
res.merge <- merge(res.lim, res.ns$table, by.x = "Name", by.y = "row.names",
                   suffixes = c(".Limma", ".NSDiff"))

colnames(res.merge)[c(7,8,12,13)] <- c("p.Limma", "q.Limma", "p.NSDiff", "q.NSDiff")

dim(res.merge)
```

```
## [1] 800  13
```

Log2FC's between the two methods are somewhat well-correlated, with a Spearman correlation of 0.7. However, NanoStringDiff calculates extremely high fold changes in some cases, generally for genes with extremely low expression in one of the two groups.

```r
ggplot(res.merge, aes(x = logFC.Limma, y = logFC.NSDiff)) +
  geom_point(alpha = 0.2)
```

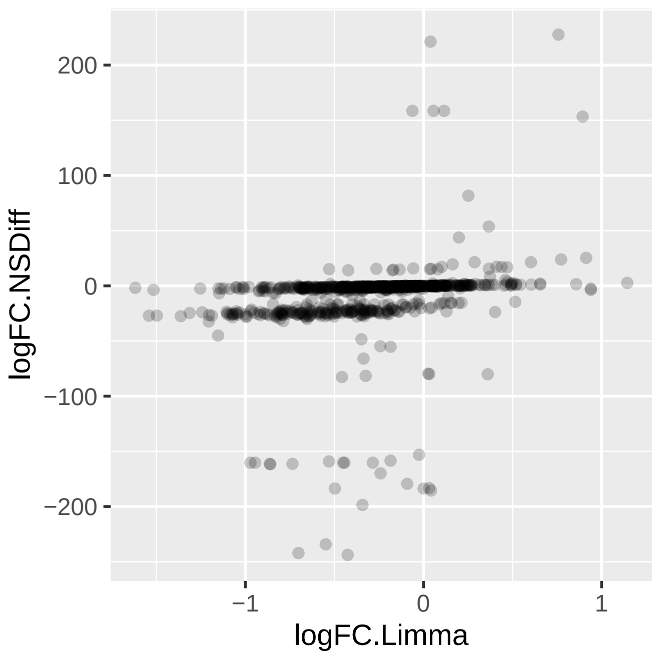


This plot zooms the y axis (log2FC from -10 to 10). The solid line shows y = x.

```
ggplot(res.merge, aes(x = logFC.Limma, y = logFC.NSDiff)) +
  geom_point(alpha = 0.2) +
  geom_abline(slope = 1, intercept = 0) +
  ylim(-10, 10)
```

```
## Warning: Removed 263 rows containing missing values (geom_point).
```

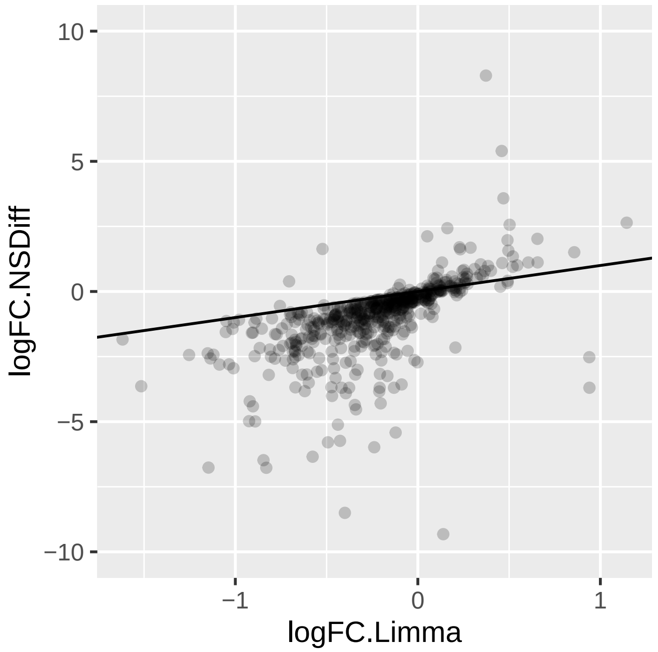


Nominal p-values are also somewhat well correlated, although NanoStringDiff generally identifies more differentially expressed genes. This is consistent with results reported by (Wang et al. 2016), where NanoStringDiff was demonstrated to have better sensitivity in simulation studies.

```
ggplot(res.merge, aes(x = -log10(p.Limma), y = -log10(p.NSDiff))) +
  geom_point(alpha = 0.2) +
  geom_abline(slope = 1, intercept = 0)
```

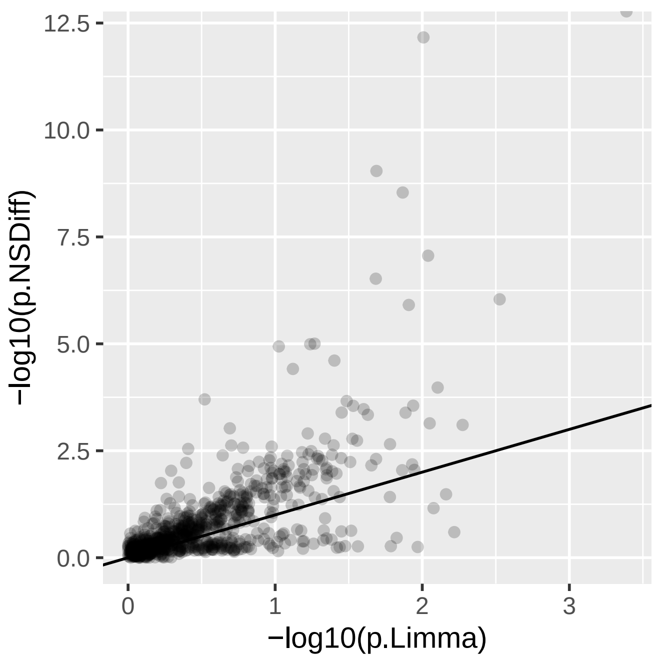


Next, we'll run Limma again, but only including genes that pass negative control check (expression is greater than the calculated noise level in at least half of samples).

```
res.lim <- runLimmaAnalysis(datDefault, base.group = "GFP-vector")

res.lim <- topTable(res.lim, number = nrow(res.lim), coef = 2)

res.less <- merge(res.lim, res.ns$table, by.x = "Name", by.y = "row.names",
                  suffixes = c(".Limma", ".NSDiff"), all = TRUE)

colnames(res.less)[c(7,8,12,13)] <- c("p.Limma", "q.Limma", "p.NSDiff", "q.NSDiff")
```

Overall, they agree much better in this case, with a Spearman correlation of 0.95.

```
ggplot(res.less[complete.cases(res.less),], aes(x = logFC.Limma, y = logFC.NSDiff)) +
  geom_point(alpha = 0.2) +
  geom_abline(slope = 1, intercept = 0)
```

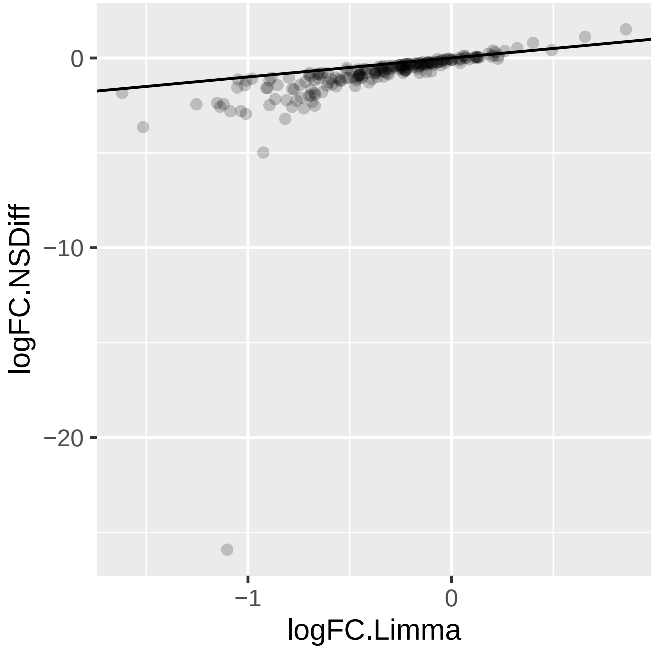


Zoomed in on the y-axis:

```
ggplot(res.less[complete.cases(res.less),], aes(x = logFC.Limma, y = logFC.NSDiff)) +
  geom_point(alpha = 0.2) +
  geom_abline(slope = 1, intercept = 0) +
  ylim(-5, 3)
```

```
## Warning: Removed 1 rows containing missing values (geom_point).
```

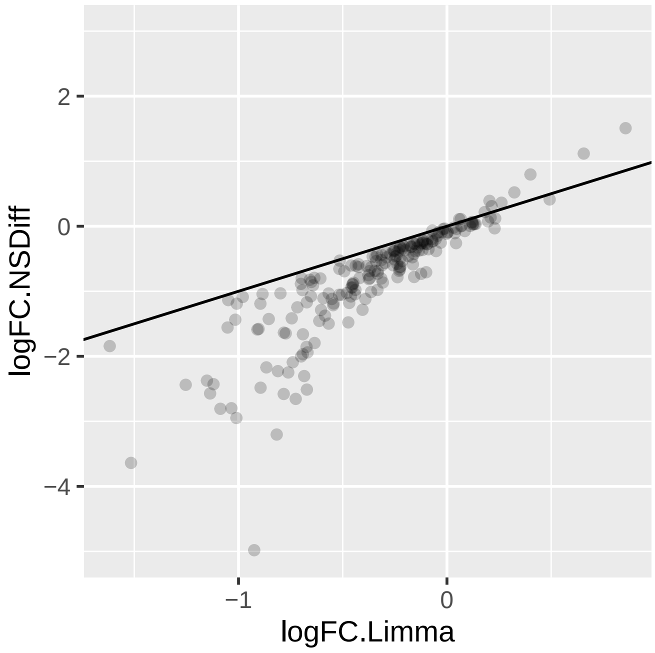


A similar pattern is seen with nominal p-values. After correcting for false discovery, 28 have q < 0.05 with NanoStringDiff, while 1 has q < 0.05 with scaling normalization and Limma.

```r
ggplot(res.less, aes(x = -log10(p.Limma), y = -log10(p.NSDiff))) +
  geom_point(alpha = 0.2) +
  geom_abline(slope = 1, intercept = 0)
```

```
## Warning: Removed 597 rows containing missing values (geom_point).
```

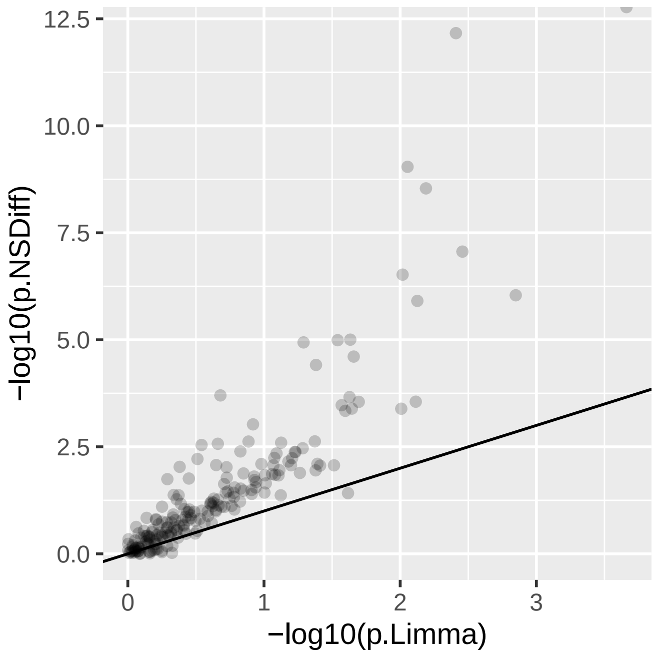


Of the top 14 DE miRNA identified by NanoStringDiff ($q < 0.01$, Table 1 in (Wang et al. 2016)), 12 were also identified as DE (nominal $p < 0.05$) by Limma analysis after standard normalization. However, only one (hsa-miR-145-5p|0) passed false discovery with $q < 0.05$.

```r
# miRNA targets in Wang et al Table 1 (q < 0.01 by NanoStringDiff)
fromPaper <- c("hsa-miR-145-5p|0", "hsa-miR-374a-5p|0", "hsa-miR-181a-5p|0",
               "hsa-miR-221-3p|0", "hsa-miR-151a-3p|0", "hsa-miR-374b-5p|0",
               "hsa-miR-152|0", "hsa-miR-29b-3p|0", "hsa-miR-130a-3p|0",
               "hsa-miR-361-5p|0", "hsa-miR-93-5p|0", "hsa-miR-143-3p|0.012",
               "hsa-miR-23b-3p|0", "hsa-miR-142-3p|0")

tab1 <- res.less[res.less$Name %in% fromPaper,]
tab1out <- tab1[order(tab1$q.NSDiff), c(1,4,7,8,10,12,13)]
tab1out[,-1] <- format(tab1out[,-1], digits = 1)

knitr::kable(tab1out,
             row.names = FALSE, align = "c")
```

| Name | logFC.Limma | p.Limma | q.Limma | logFC.NSDiff | p.NSDiff | q.NSDiff |
|---|---|---|---|---|---|---|
| hsa-miR-145-5p\|0 | -1.6 | 2e-04 | 0.04 | -1.8 | 0e+00 | 0e+00 |
| hsa-miR-374a-5p\|0 | -1.0 | 4e-03 | 0.20 | -1.2 | 7e-13 | 3e-10 |
| hsa-miR-181a-5p\|0 | -0.9 | 9e-03 | 0.20 | -1.0 | 9e-10 | 2e-07 |
| hsa-miR-221-3p\|0 | -1.0 | 6e-03 | 0.20 | -1.1 | 3e-09 | 6e-07 |
| hsa-miR-151a-3p\|0 | -1.0 | 3e-03 | 0.20 | -1.4 | 9e-08 | 1e-05 |
| hsa-miR-374b-5p\|0 | -0.9 | 1e-02 | 0.20 | -1.2 | 3e-07 | 4e-05 |
| hsa-miR-152\|0 | -1.3 | 1e-03 | 0.14 | -2.4 | 9e-07 | 1e-04 |

| Name | logFC.Limma | p.Limma | q.Limma | logFC.NSDiff | p.NSDiff | q.NSDiff |
|---|---|---|---|---|---|---|
| hsa-miR-29b-3p\|0 | -1.0 | 7e-03 | 0.20 | -1.1 | 1e-06 | 1e-04 |
| hsa-miR-130a-3p\|0 | -0.7 | 3e-02 | 0.31 | -0.9 | 1e-05 | 8e-04 |
| hsa-miR-361-5p\|0 | -0.7 | 2e-02 | 0.30 | -1.0 | 1e-05 | 8e-04 |
| hsa-miR-93-5p\|0 | -0.6 | 5e-02 | 0.39 | -0.8 | 1e-05 | 8e-04 |
| hsa-miR-143-3p\|0.012 | -0.8 | 2e-02 | 0.30 | -1.0 | 2e-05 | 2e-03 |
| hsa-miR-23b-3p\|0 | -0.7 | 4e-02 | 0.34 | -0.8 | 4e-05 | 2e-03 |
| hsa-miR-142-3p\|0 | NA | NA | NA | 2.6 | 1e-04 | 6e-03 |

```
# QC check OK- NanoStringDiff statistics here match those presented in Wang et al.
```

4 non-DE miRNA (hsa-miR-155, hsa-miR-106, hsa-miR-9, hsa-miR-210) identified and validated by qPCR in the paper were clearly non-DE by both methods in this case.

## A larger data set

We did a similar comparison with data set GSE117751 (Lundy et al. 2018), which compared mRNA expression from blood samples of 14 patients with autoimmune retinopathy vs. 14 healthy controls (there was also a set of patients with retinitis pigmentosa, which are not considered in this vignette).

```
rcc_data <- system.file("extdata", "GSE117751_RAW", package = "NanoTube")
sample_data <- system.file("extdata", "GSE117751_sample_data.csv",
                           package = "NanoTube")

# No normalization, to use in NanoStringDiff
datNoNorm <- processNanostringData(rcc_data,
                             sampleTab = sample_data,
                             idCol = "GEO_Accession", groupCol = "Sample_Diagnosis",
                             normalization = "none")

# Use scaling normalization for positive and housekeeping controls,
# but do not use negative control genes.
datKeep <- processNanostringData(rcc_data,
                             sampleTab = sample_data,
                             idCol = "GEO_Accession", groupCol = "Sample_Diagnosis",
                             bgProportion = 0) # keep all genes

# Use default scaling normalization and removal of genes that do
# not exceed "noise" level in at least half of the samples.
datDefault <- processNanostringData(rcc_data,
                             sampleTab = sample_data,
                             idCol = "GEO_Accession", groupCol = "Sample_Diagnosis",)

# Provides basic QC parameters
datQC <- processNanostringData(rcc_data,
                             sampleTab = sample_data,
                             idCol = "GEO_Accession", groupCol = "Sample_Diagnosis",
                             output.format = "list")
```

Positive control scale factors are ok.

```r
summary(datQC$pc.scalefactors)
```

```
##    Min. 1st Qu.  Median    Mean 3rd Qu.    Max. 
##  0.5505  0.6915  0.8393  1.0000  1.1325  2.2336
```

Housekeeping scale factors are also ok.

```r
summary(datQC$hk.scalefactors)
```

```
##    Min. 1st Qu.  Median    Mean 3rd Qu.    Max. 
##  0.4357  0.6747  0.9812  1.0000  1.2294  1.9085
```

20-30% of genes in this data set are expressed at a lower level than "background", which is calculated as the mean plus two standard deviations of the negative controls. 130 of 608 genes are discarded from this data set with the default filtering.

```r
knitr::kable(signif(datQC$bg.stats, digits = 2),
             row.names = TRUE, align = "c")
```

| | Mean.Neg | Max.Neg | sd.Neg | background | num.less.bg | frc.less.bg |
|---|---|---|---|---|---|---|
| GSM3308226_20160715_5609WV-C.RCC | 6.1 | 14.0 | 5.6 | 17.0 | 120 | 0.21 |
| GSM3308227_20170504_6090AA-A2.RCC | 11.0 | 18.0 | 5.6 | 22.0 | 140 | 0.23 |
| GSM3308228_20170504_6121PF-A2.RCC | 14.0 | 31.0 | 8.8 | 31.0 | 150 | 0.27 |
| GSM3308229_20170504_5993KP-A2.RCC | 10.0 | 21.0 | 6.0 | 23.0 | 150 | 0.26 |
| GSM3308230_20170504_6536VE-A2.RCC | 10.0 | 16.0 | 3.5 | 17.0 | 150 | 0.25 |
| GSM3308231_20170504_6568NC-A2.RCC | 10.0 | 24.0 | 7.5 | 25.0 | 170 | 0.29 |
| GSM3308232_20170504_6612MK-A2.RCC | 12.0 | 29.0 | 8.3 | 29.0 | 170 | 0.29 |
| GSM3308233_20170504_6636KS-A2.RCC | 13.0 | 21.0 | 5.5 | 24.0 | 160 | 0.28 |
| GSM3308234_20170504_6204CL-A2.RCC | 10.0 | 25.0 | 7.6 | 26.0 | 160 | 0.28 |
| GSM3308235_20161006_6779BL-B.RCC | 13.0 | 28.0 | 8.1 | 29.0 | 130 | 0.23 |
| GSM3308236_20161006_4988CH-A.RCC | 9.5 | 15.0 | 5.0 | 19.0 | 120 | 0.21 |
| GSM3308237_20160329_8017DU-A.RCC | 6.4 | 14.0 | 3.9 | 14.0 | 130 | 0.23 |
| GSM3308238_20160715_9154ET-A.RCC | 8.5 | 22.0 | 8.3 | 25.0 | 68 | 0.12 |
| GSM3308239_20160715_9164SM-A.RCC | 5.2 | 14.0 | 4.1 | 13.0 | 120 | 0.20 |
| GSM3308240_20161006_3947MC-A.RCC | 13.0 | 28.0 | 8.9 | 31.0 | 140 | 0.24 |
| GSM3308241_20160629_402ER-A.RCC | 7.6 | 19.0 | 7.2 | 22.0 | 130 | 0.22 |
| GSM3308242_20160629_991KH-A.RCC | 8.0 | 15.0 | 5.3 | 19.0 | 130 | 0.23 |
| GSM3308243_20160715_440JT-B.RCC | 6.7 | 13.0 | 4.1 | 15.0 | 110 | 0.20 |

| | Mean.Neg | Max.Neg | sd.Neg | background | num.less.bg | frc.less.bg |
|---|---|---|---|---|---|---|
| GSM3308244_20160629_5092AD-A.RCC | 7.7 | 18.0 | 6.6 | 21.0 | 140 | 0.23 |
| GSM3308245_20170504_8037AM-A3.RCC | 8.9 | 24.0 | 7.8 | 24.0 | 150 | 0.26 |
| GSM3308246_20170911_3905SW-A.RCC | 7.4 | 13.0 | 3.7 | 15.0 | 120 | 0.21 |
| GSM3308247_20160629_4231DK-A.RCC | 8.6 | 22.0 | 7.4 | 23.0 | 120 | 0.22 |
| GSM3308248_20160628_6372ML-A.RCC | 11.0 | 28.0 | 9.9 | 31.0 | 120 | 0.22 |
| GSM3308249_20170911_5665PB-A.RCC | 5.9 | 11.0 | 3.8 | 13.0 | 110 | 0.18 |
| GSM3308250_20170911_9134LH-B.RCC | 7.4 | 13.0 | 3.7 | 15.0 | 110 | 0.18 |
| GSM3308251_20160715_6034RN-A.RCC | 6.1 | 16.0 | 5.0 | 16.0 | 120 | 0.21 |
| GSM3308252_20161006_9219RD-A.RCC | 11.0 | 24.0 | 8.2 | 27.0 | 140 | 0.25 |
| GSM3308253_20161005_2068AP-A.RCC | 12.0 | 27.0 | 8.7 | 30.0 | 150 | 0.25 |
| GSM3308254_20170504_5885SL-D2.RCC | 9.5 | 18.0 | 5.5 | 21.0 | 140 | 0.25 |
| GSM3308255_20170504_9051LS-A3.RCC | 6.5 | 10.0 | 3.1 | 13.0 | 130 | 0.22 |
| GSM3308256_20160628_100KB-A2.RCC | 9.2 | 19.0 | 6.3 | 22.0 | 130 | 0.23 |
| GSM3308257_20160628_9149DB-A.RCC | 7.6 | 20.0 | 6.4 | 20.0 | 130 | 0.22 |
| GSM3308258_20160628_9147BM-A.RCC | 8.1 | 20.0 | 5.9 | 20.0 | 120 | 0.21 |
| GSM3308259_20160628_9148CW-A.RCC | 5.7 | 16.0 | 5.6 | 17.0 | 120 | 0.21 |
| GSM3308260_20161005_9281JM-A.RCC | 11.0 | 26.0 | 9.1 | 29.0 | 140 | 0.24 |
| GSM3308261_20161005_133BK-A.RCC | 11.0 | 21.0 | 7.1 | 25.0 | 140 | 0.24 |
| GSM3308262_20161005_134CD-A.RCC | 14.0 | 28.0 | 8.3 | 30.0 | 130 | 0.22 |
| GSM3308263_20170911_9653RA-A.RCC | 6.5 | 15.0 | 4.3 | 15.0 | 140 | 0.24 |
| GSM3308264_20170911_9655RK-A.RCC | 7.4 | 18.0 | 4.5 | 16.0 | 140 | 0.24 |
| GSM3308265_20170911_9656CW-A.RCC | 9.0 | 14.0 | 3.4 | 16.0 | 130 | 0.23 |
| GSM3308266_20170911_9654NL-A.RCC | 5.5 | 8.3 | 1.8 | 9.2 | 86 | 0.15 |
| GSM3308267_20170911_9660TM-A.RCC | 5.9 | 11.0 | 2.9 | 12.0 | 140 | 0.24 |

Conduct Limma analysis with the default scaling options and no usage of negative control genes. Again,

the low expression genes are not expected to conform to a normal distribution, so this is not generally recommended. In this case, including all genes will allow us to compare results of all genes between the two methods. As expected, Limma analysis was still quite fast on an HP laptop with 2 x 16 MB memory.

```
start_time <- Sys.time()

res.lim <- runLimmaAnalysis(datKeep, base.group = "None")

end_time <- Sys.time()
signif(end_time - start_time, digits = 1)
```

```
## Time difference of 0.03 secs
```

```
# Retain results for Autoimmune Retinopathy vs. Control
res.lim <- topTable(res.lim, number = nrow(res.lim), coef = 2)
```

Prepare the data set for NanoStringDiff analysis.

```
dat.ns <- makeNanoStringSetFromEset(datNoNorm)

dat.ns <- NanoStringDiff::estNormalizationFactors(dat.ns)

knitr::kable(head(pData(dat.ns)),
             row.names = TRUE, align = "c")
```

| | Autoimmune retinopathy | None | Retinitis pigmentosa |
|---|---|---|---|
| GSM3308226_20160715_5609WV-C.RCC | 1 | 0 | 0 |
| GSM3308227_20170504_6090AA-A2.RCC | 1 | 0 | 0 |
| GSM3308228_20170504_6121PF-A2.RCC | 1 | 0 | 0 |
| GSM3308229_20170504_5993KP-A2.RCC | 1 | 0 | 0 |
| GSM3308230_20170504_6536VE-A2.RCC | 1 | 0 | 0 |
| GSM3308231_20170504_6568NC-A2.RCC | 1 | 0 | 0 |

Run NanoStringDiff analysis. This was not run on the laptop, but on a GPU cluster where the following analysis took 1.6 hours.

```
# Not run on laptop

start_time <- Sys.time()

res.ns <- NanoStringDiff::glm.LRT(dat.ns,
                                  design.full = as.matrix(pData(dat.ns)),
                                  contrast = c(1, -1, 0))  # AR vs. None
end_time <- Sys.time()
end_time - start_time

saveRDS(res.ns, "GSE117751_nsDiffRes-AR.rds")
```

```r
res.ns <- readRDS("../../extra_data/GSE117751_nsDiffRes-AR.rds")

nsTab <- head(res.ns$table[order(res.ns$table$pvalue),])
nsTab[,1:4] <- lapply(nsTab, format, digits = 1, nsmall = 1)

knitr::kable(nsTab,
             row.names = TRUE, align = "c")
```

| | logFC | lr | pvalue | qvalue |
|---|---|---|---|---|
| IKBKB | 0.5 | 25.7 | 4e-07 | 1e-04 |
| EGR1 | 23.3 | 24.7 | 7e-07 | 1e-04 |
| CXCL9 | 22.9 | 24.2 | 9e-07 | 1e-04 |
| IFNA2 | 21.8 | 23.8 | 1e-06 | 1e-04 |
| CXCL11 | 1.7 | 23.7 | 1e-06 | 1e-04 |
| IL8 | -1.3 | 21.1 | 4e-06 | 4e-04 |

Merge the data tables from the two analyses.

```r
res.merge <- merge(res.lim, res.ns$table, by.x = "Name", by.y = "row.names",
                   suffixes = c(".Limma", ".NSDiff"))

colnames(res.merge)[c(7,8,12,13)] <- c("p.Limma", "q.Limma", "p.NSDiff", "q.NSDiff")

dim(res.merge)
```

```
## [1] 579  13
```

Log2FC's between the two methods are again somewhat well-correlated, with a Spearman correlation of 0.6. Again, NanoStringDiff calculates extremely high fold changes in some cases, generally for genes with extremely low expression in one of the two groups.

```r
ggplot(res.merge, aes(x = logFC.Limma, y = logFC.NSDiff)) +
  geom_point(alpha = 0.2)
```

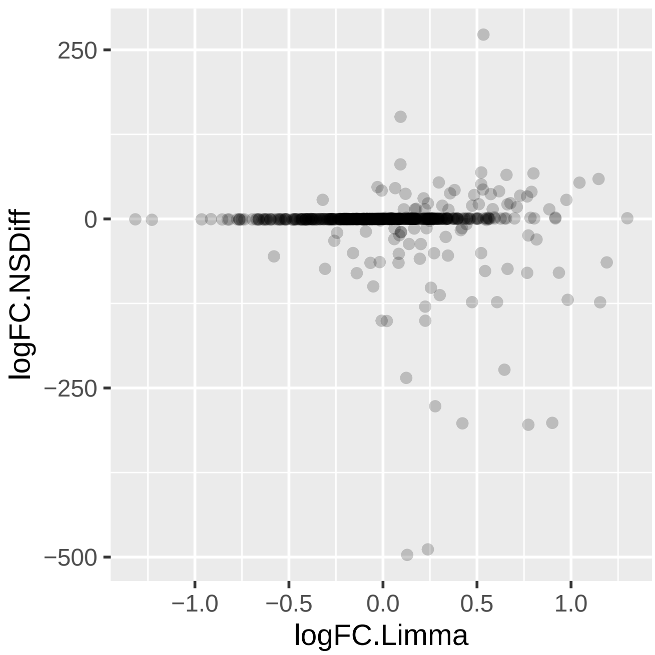


This plot zooms the y axis (log2FC for NanoStringDiff from -5 to 5), to demonstrate that very similar fold changes are calculated in most cases. The line shows y = x.

```
ggplot(res.merge, aes(x = logFC.Limma, y = logFC.NSDiff)) +
  geom_point(alpha = 0.2) +
  geom_abline(slope = 1, intercept = 0) +
  ylim(-5, 5)
```

```
## Warning: Removed 94 rows containing missing values (geom_point).
```

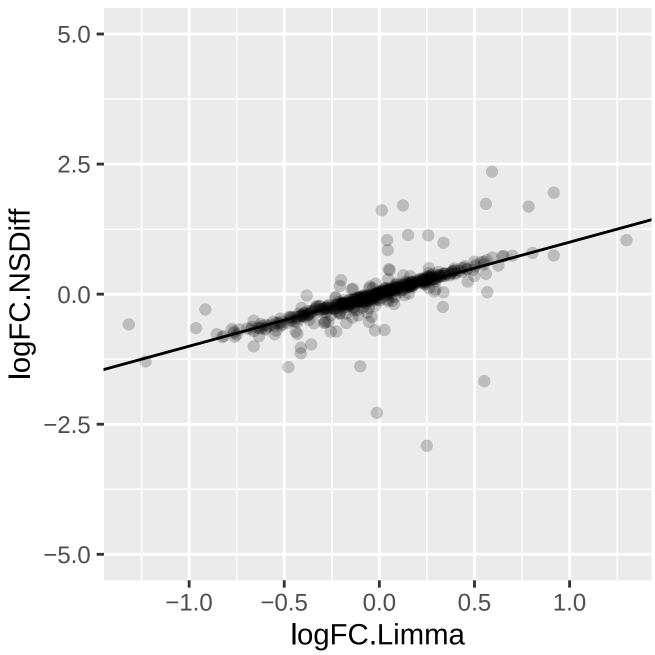


Nominal p-values are also reasonably well correlated, although NanoStringDiff generally identifies more differentially expressed genes. However, the difference between the two methods is not nearly as extreme as it

was in the first data set.

```
ggplot(res.merge, aes(x = -log10(p.Limma), y = -log10(p.NSDiff))) +
  geom_point(alpha = 0.2) +
  geom_abline(slope = 1, intercept = 0)
```

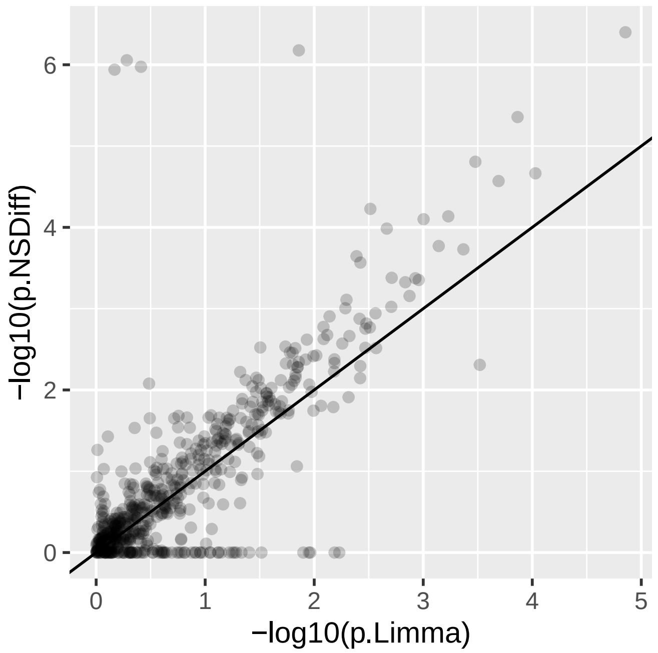


Next, we'll again run Limma, only keeping genes that pass negative control check.

```
res.lim <- runLimmaAnalysis(datDefault, base.group = "None")

res.lim <- topTable(res.lim, number = nrow(res.lim), coef = 2)

res.less <- merge(res.lim, res.ns$table, by.x = "Name", by.y = "row.names",
                  suffixes = c(".Limma", ".NSDiff"), all = TRUE)

colnames(res.less)[c(7,8,12,13)] <- c("p.Limma", "q.Limma", "p.NSDiff", "q.NSDiff")
```

After filtering low-expressed genes, the two methods agree quite well with a Spearman correlation of 0.95.

```
ggplot(res.less[complete.cases(res.less),], aes(x = logFC.Limma, y = logFC.NSDiff)) +
  geom_point(alpha = 0.2) +
  geom_abline(slope = 1, intercept = 0)
```

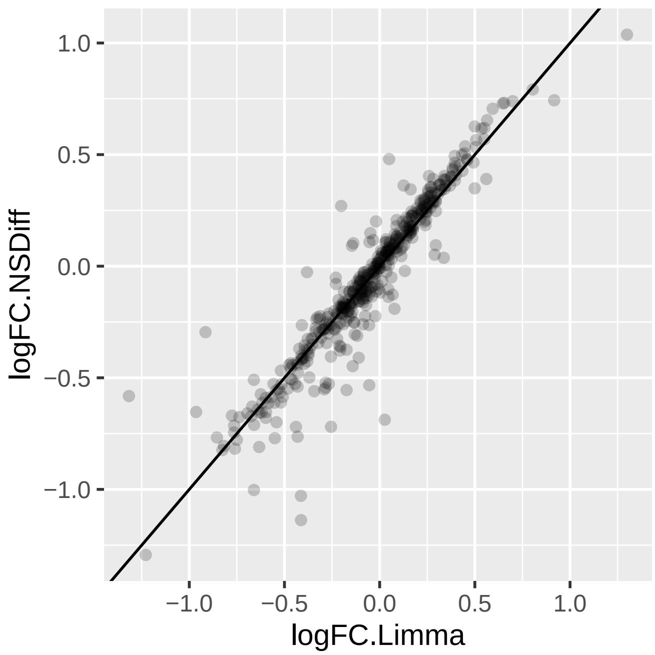


A similar pattern is seen with nominal p-values. After correcting for false discovery, 46 have $q < 0.05$ with NanoStringDiff, while 13 have $q < 0.05$ with scaling normalization and Limma.

```
ggplot(res.less, aes(x = -log10(p.Limma), y = -log10(p.NSDiff))) +
  geom_point(alpha = 0.2) +
  geom_abline(slope = 1, intercept = 0)
```

```
## Warning: Removed 130 rows containing missing values (geom_point).
```

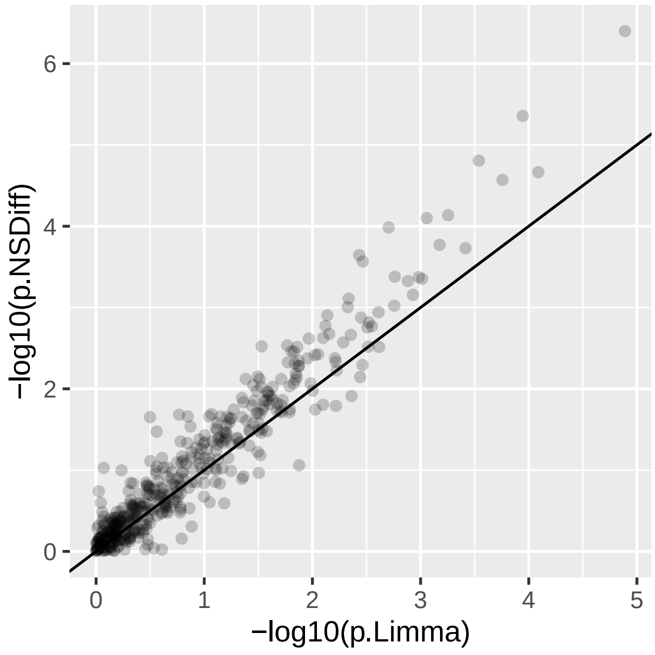


The results in this section generally agree with those reported by (Wang et al. 2016). NanoStringDiff still identifies more differentially expressed genes than the traditional scaling method in large sample sizes, but the difference between the two methods is significantly smaller. Additionally, NanoStringDiff is quite

computationally intense in this case. Additional validation would be important to confirm that the genes identified by either or both methods are indeed differentially expressed.

# Recommendations

NanoStringDiff was developed to account for three challenges with NanoString data:

1. The analysis of count data, which had been rather inappropriately done using t-test type methods.
2. The inclusion of positive control and housekeeping size factors.
3. The consideration of background noise, which is not explicitly handled by RNA-seq-based methods.

For analyses with very small sample sizes (preferably n less than 5), NanoStringDiff has been demonstrated to perform better than other analysis methods, particularly when there are many low expression genes which would be poorly handled (or filtered out) by the standard NanoString normalization & Limma analysis. With a larger sample size and more counts per gene, NanoStringDiff becomes much slower and potentially unworkable on a standard computer. Additionally, the advantage in sensitivity detecting differentially expressed genes has been demonstrated to be less substantial with larger sample sizes (Wang et al. 2016). In this case, Limma is likely to perform reasonably well in a much shorter time, or NanoStringDiff can be run using high-performance computing resources.

# Session Info

```
sessionInfo()
```

```
## R version 4.1.2 (2021-11-01)
## Platform: x86_64-w64-mingw32/x64 (64-bit)
## Running under: Windows 10 x64 (build 19042)
##
## Matrix products: default
##
## locale:
## [1] LC_COLLATE=English_United States.1252
## [2] LC_CTYPE=English_United States.1252
## [3] LC_MONETARY=English_United States.1252
## [4] LC_NUMERIC=C
## [5] LC_TIME=English_United States.1252
##
## attached base packages:
## [1] stats     graphics  grDevices utils     datasets  methods   base
##
## other attached packages:
## [1] NanoTube_1.3.6      limma_3.53.3        ggplot2_3.3.6
## [4] Biobase_2.57.1      BiocGenerics_0.43.0
##
## loaded via a namespace (and not attached):
##  [1] tidyselect_1.1.2      fgsea_1.23.0          xfun_0.29
##  [4] purrr_0.3.4           lattice_0.20-45       colorspace_2.0-3
##  [7] vctrs_0.4.1           generics_0.1.2        htmltools_0.5.2
## [10] yaml_2.2.2            utf8_1.2.2            rlang_1.0.2
## [13] pillar_1.7.0          glue_1.6.1            withr_2.5.0
## [16] DBI_1.1.3             BiocParallel_1.31.8   matrixStats_0.62.0
## [19] lifecycle_1.0.1       plyr_1.8.7            stringr_1.4.0
## [22] munsell_0.5.0         gtable_0.3.0          codetools_0.2-18
## [25] evaluate_0.15         labeling_0.4.2        knitr_1.39
```

```
## [28] fastmap_1.1.0         parallel_4.1.2       fansi_1.0.2
## [31] highr_0.9             Rcpp_1.0.8.3         scales_1.2.0
## [34] farver_2.1.0          gridExtra_2.3        fastmatch_1.1-3
## [37] digest_0.6.29         stringi_1.7.6        dplyr_1.0.9
## [40] grid_4.1.2            cli_3.3.0            tools_4.1.2
## [43] magrittr_2.0.1        tibble_3.1.6         crayon_1.5.1
## [46] pkgconfig_2.0.3       ellipsis_0.3.2       Matrix_1.3-4
## [49] data.table_1.14.2     assertthat_0.2.1     rmarkdown_2.14
## [52] reshape_0.8.9         rstudioapi_0.13      R6_2.5.1
## [55] NanoStringDiff_1.27.0 compiler_4.1.2
```

# References


Lundy, Steven K., Enayat Nikoopour, Athanasios J. Karoukis, Ray Ohara, Mohammad I. Othman, Rebecca Tagett, K. Thiran Jayasundera, and John R. Heckenlively. 2018. "T Helper 1 Cellular Immunity Toward Recoverin Is Enhanced in Patients With Active Autoimmune Retinopathy." *Frontiers in Medicine* 5: 249. https://doi.org/10.3389/fmed.2018.00249.

Ritchie, Matthew E., Belinda Phipson, Di Wu, Yifang Hu, Charity W. Law, Wei Shi, and Gordon K. Smyth. 2015. "Limma Powers Differential Expression Analyses for RNA-Sequencing and Microarray Studies." *Nucleic Acids Research* 43 (7): e47.

Waggott, Daryl, Kenneth Chu, Shaoming Yin, Bradly G. Wouters, Fei-Fei Liu, and Paul C. Boutros. 2012. "NanoStringNorm: An Extensible R Package for the Pre-Processing of NanoString mRNA and miRNA Data." *Bioinformatics* 28 (11): 1546–48. https://doi.org/10.1093/bioinformatics/bts188.

Wang, Hong, Craig Horbinski, Hao Wu, Yinxing Liu, Shaoyi Sheng, Jinpeng Liu, Heidi Weiss, Arnold J. Stromberg, and Chi Wang. 2016. "NanoStringDiff: A Novel Statistical Method for Differential Expression Analysis Based on NanoString nCounter Data." *Nucleic Acids Res* 44 (20): e151–51. https://doi.org/10.1093/nar/gkw677.
